# Artificial Urinary Sphincter in a High-Risk Urethra: Transcorporal Gullwing Modification Description of the Technique

**DOI:** 10.5152/tud.2022.22134

**Published:** 2022-11-01

**Authors:** David Hernández-Hernández, María Yanira Ortega-González, Bárbara Padilla-Fernández, David Manuel Castro-Díaz

**Affiliations:** 1 Department of Urology, University Hospital of the Canary Islands, Tenerife, Spain; 2 Department of Surgery, Universidad de La Laguna Faculty of Medicine, Tenerife, Spain

**Keywords:** Artificial urinary sphincter, stress urinary incontinence, reoperation, revision surgery, urethra

## Abstract

**Background:**

In this report, we describe a modification of transcorporal artificial urinary sphincter placement known as Gullwing modification.

**Description of Technique:**

Using a penoscrotal approach, bilateral corpora cavernosa flaps are harvested and sutured in the midline covering the lateral and ventral surfaces of the urethra. Transcorporal cuff placement provides dorsal reinforcement, thus having extra tissue buttressing all the circumference in cases of a fragile urethra due to previous urethral cuff erosion, urethroplasty, or pelvic radiotherapy.

**Patient and Methods:**

After previous urethral cuff erosion, radiotherapy, and urethral reconstruction, our patient complained of severe stress urinary incontinence. Due to the high risk of urethral complications, we proceed to a transcorporal artificial sphincter placement with urethral reinforcement through a bilateral cavernosal flap.

**Results:**

The surgery was successfully completed, and after 6 weeks, sphincter was activated with satisfactory results. Two years after surgery, his continence status is stable without complications.

**Conclusion:**

Urethral complications associated with artificial urinary sphincter surgery remain a challenge for the reconstructive surgeon. Reinforcement of the ventral aspect of the urethra through corpora cavernosal flaps may reduce the likelihood of urethral erosion in high-risk cases.

## Background

The artificial urinary sphincter (AUS) remains the gold standard in the treatment of severe male stress urinary incontinence. With more than 30 years of experience, AUS offers consistent results (61%-100% of social continence rates), with a good balance between efficacy and complications.^1^ Reoperation rate is probably the main drawback of AUS, with around 50% of patients requiring revision in 10 years because of malfunction, infection, or erosion.^1,2^

Different factors have been associated with the higher risk of revision surgery, especially those related to the “fragile or high-risk urethra”: prior radiotherapy, history of failed AUS, and/or previous urethroplasty.^3-5^

This group of patients represents a challenge for the surgeon, especially if these negative factors coexist. The aim of this article is to describe a novel modification of the transcorporal approach that could add some benefits to the extremely fragile urethra.

## Description of Technique

**Figure 1.**
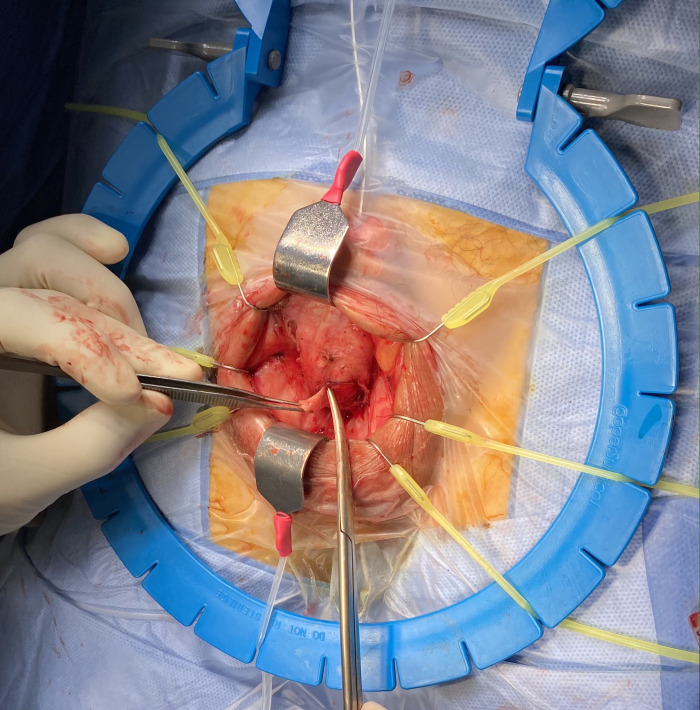
Bilateral corpora cavernosa flap harvesting.

**Figure 2.**
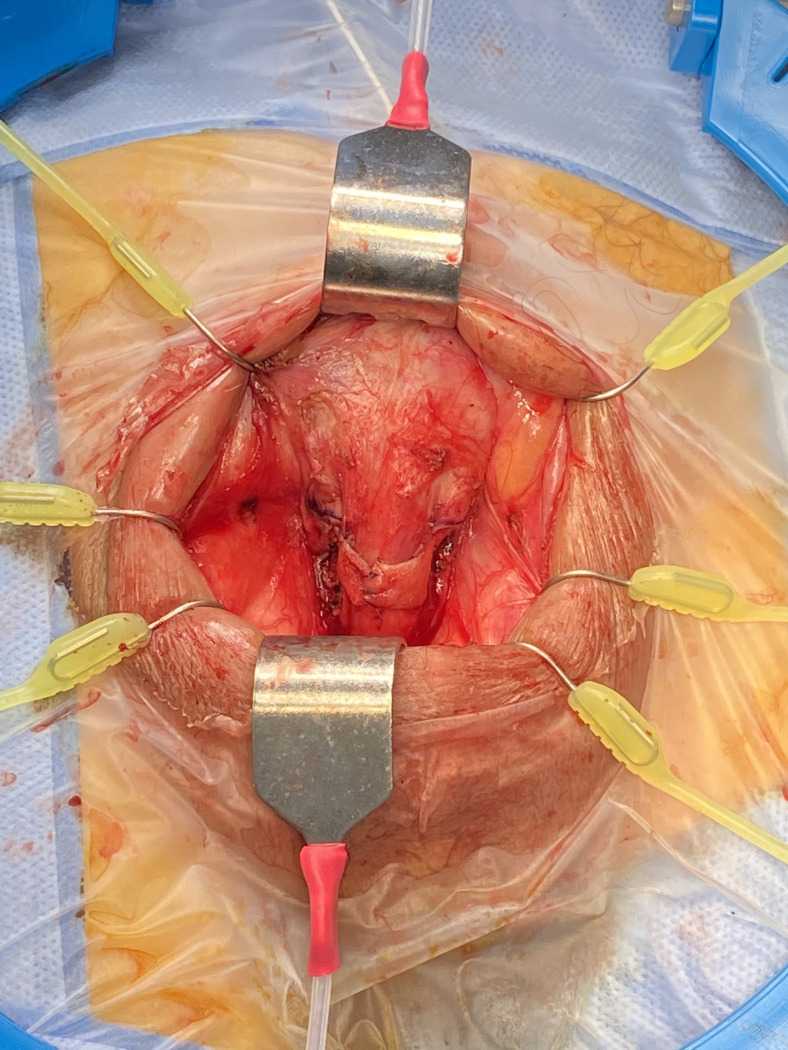
Bilateral corpora cavernosa sutured in the midline.

**Figure 3.**
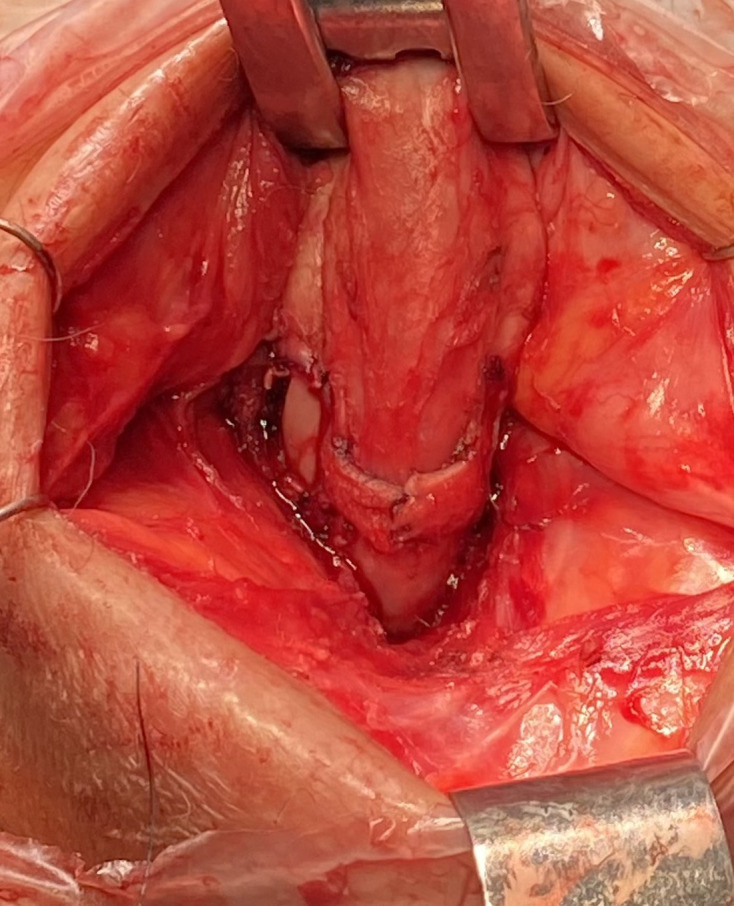
Bovine pericardium flap covering cavernosal defect.

Due to the high risk of erosion or intraoperative urethral injury, we decided to perform a transcorporal AUS placement adding cavernosal tunica albuginea flaps (transcorporal Gullwing modification),^6^ but due to previous urethroplasty with crural separation, we did a penoscrotal approach instead of a regular perineal approach. In low lithotomy position, the genitalia are scrubbed with povidone-iodine soap for 10 minutes and preoperative antibiotic prophylaxis was administered. A 16 F transurethral catheter is placed to empty the bladder. Next, a 5 cm transverse incision at the level of the penoscrotal junction is made, followed by dissection through the tunica dartos up to the urethra. Rectangular 1 cm length and width flaps are harvested bilaterally from the albuginea from the corpora cavernosa (Figure 1). These flaps are sutured ventrally covering the ventrolateral aspect of the urethra, as shown in Figure 2. Cavernosal defect is closed with a patch of bovine pericardium with running 4/0 polyglactin suture (Figure 3), and afterward, AUS cuff sizing is performed over the flap-reinforced urethra. 

## Patients and Methods

A 70-year-old male underwent laparoscopic radical prostatectomy in 2010 because of a pT3a ISUP 3 prostate cancer. As complications, he suffered from mild erectile dysfunction and stress urinary incontinence and was managed conservatively. Six months after surgery, he started to complain of weak stream and dysuria. Cystoscopy and cystourethrography revealed a short (1-1.5 cm) proximal bulbar stricture and was managed endoscopically.

Three years after prostate surgery, he developed biochemical recurrence and was treated with salvage radiotherapy and a short course of androgen deprivation therapy. Subsequently, erectile dysfunction and urinary incontinence worsened and the patient demanded surgical treatment. 

**Figure 4.**
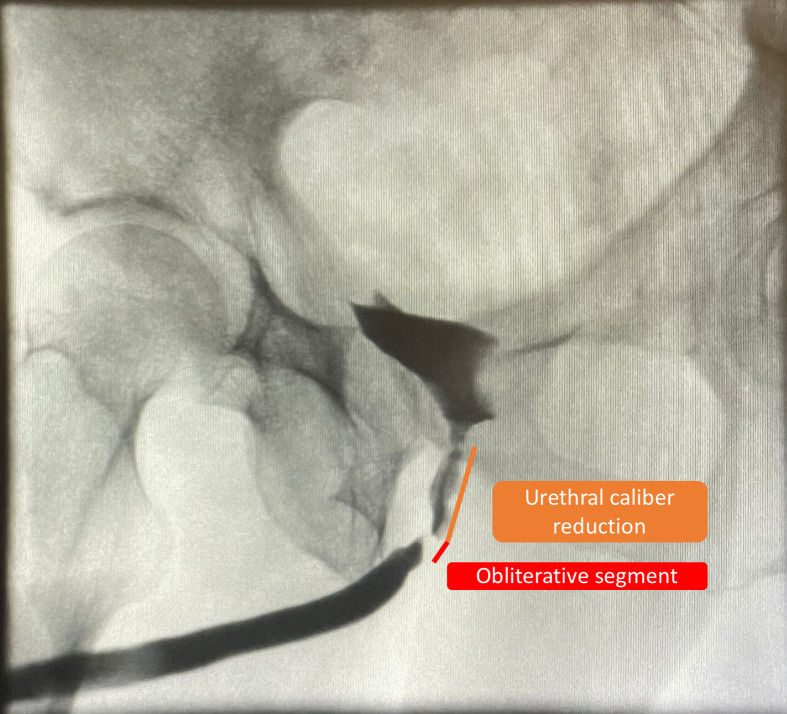
Urethrogram: bulbar urethral stricture.

After preoperative evaluation, an artificial sphincter AMS-800 was placed in a standard fashion in September 2014 with good results. Unfortunately, 1 year later, the patient developed cuff erosion requiring AUS removal and urethral repair by simple suture approximation over a 16 F catheter. The catheter was removed 2 weeks after surgery, and the patient returned to his previous incontinence status. However, a few weeks later, he started to complain of pain during micturition, increased frequency, and weak urine stream. Retrograde urethrogram showed a short segment of almost-obliterated proximal bulbar urethra with additional 2-3 cm of proximal caliber reduction (Figure 4).

**Figure 5.**
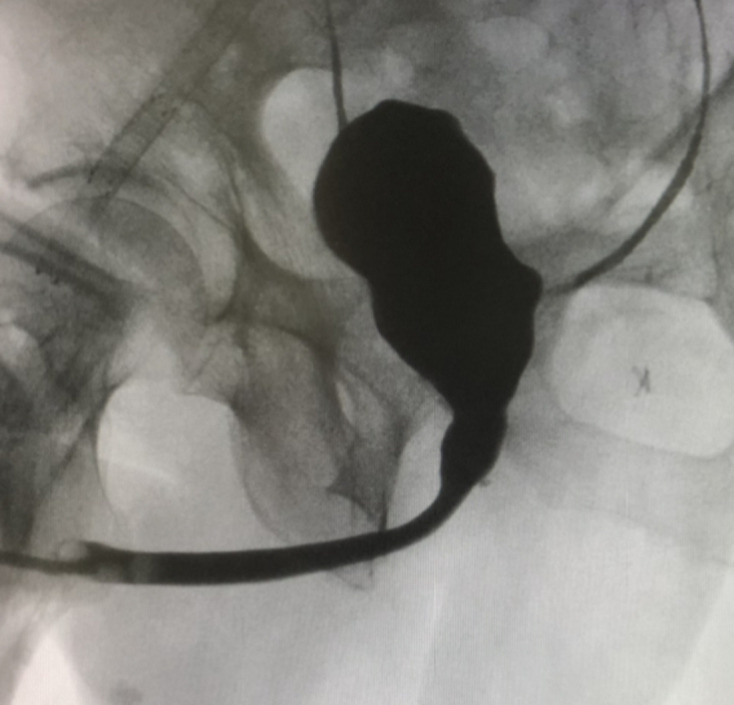
Urethrogram 6 months after urethral reconstruction.

Treatment options were discussed (endoscopic treatment associated with chronic self-dilatations vs. open urethral reconstruction) and we decided to perform anastomotic urethroplasty. Urethral narrowing was approximately 4 cm long, so wide urethral mobilization and crural separation were needed to perform anastomotic urethroplasty without tension. Postoperative course was uneventful and the outcome of urethral reconstruction was satisfactory as the 6-month postoperative retrograde urethrogram as shown in Figure 5. However, urinary incontinence persisted, so we decided to perform a second AUS placement using a penoscrotal approach and the Gullwing modification of transcorporal technique as previously described. 

## Results

**Figure 6.**
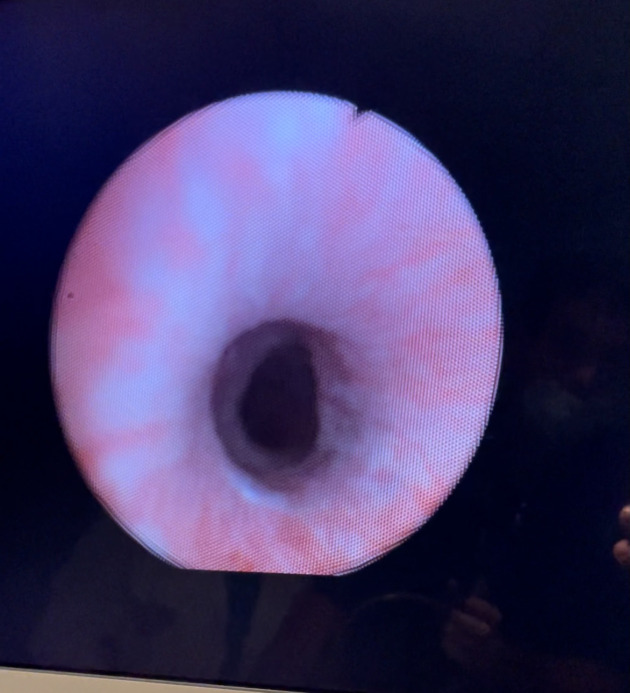
Urethroscopy 6 months after artificial sphincter placement: good urethral caliber.

**Figure 7.**
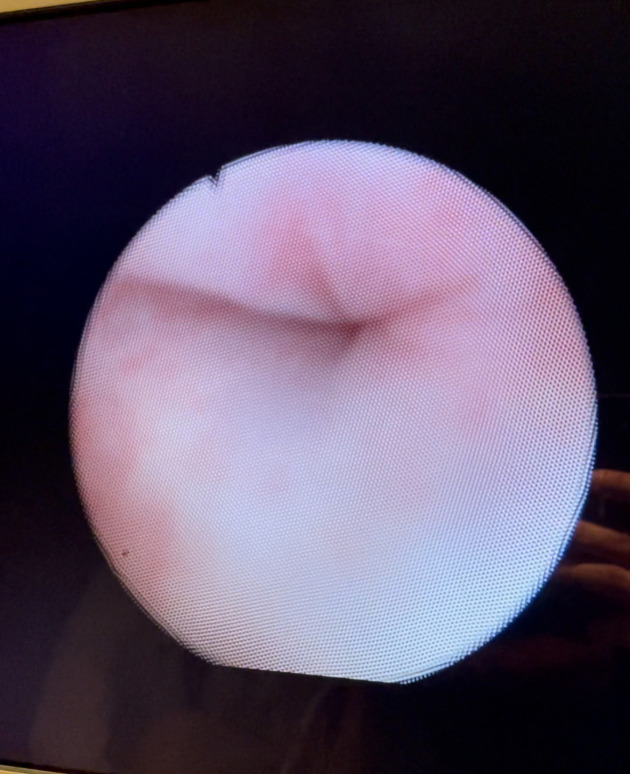
Urethroscopy 6 months after artificial sphincter placement: good sphincter coaptation.

Postoperative course was uneventful, with catheter removal 48 hours after surgery to prevent urinary retention, as opposed to 12-24 hours which is our regular practice in non-complicated AUS placement. Six weeks after surgery, the AUS was activated with very satisfactory results (going from 5-6 pads/day to 1 pad every 1-2 days). Six months after AUS placement and within our follow-up schedule of urethral stricture surgery, we performed urethrocystoscopy, confirming good urethral patency and AUS coaptation (Figures 6 and 7).

Two years after surgery, the patient maintains a good continence status (0-1 pad/day) and no urethral stricture recurrence occurred. We offered penile prosthesis implant to deal with erectile dysfunction, but due to his favorable continence status and taking into account the risk of complications, the patient refused.

## Discussion

Artificial urinary sphincter is the current gold standard for severe stress urinary incontinence.^1^ Although its efficacy is high, with a dry rate of around 58% after 3 years and a social continence rate (defined as the use of ≤1pad/day) even higher, the risk of complications requiring revision (infection, erosion, or malfunction) is still high.^1,2^ Patients with fragile urethra due to radiotherapy, previous surgeries, or atrophy have a higher complication rate, especially erosion and poor urethral coaptation.^3^ Different techniques have been implemented in order to decrease the odds of complications in these higher-risk patients: placing a more proximal or distal cuff, positioning 2 cuffs in tandem, or buttressing the urethra, either with an intestinal submucosa wrap, with a fibrin-coated collagen fleece, or with the albuginea of the corpora cavernosa.^4,5,7^

Nelson first described in 1986 the use of the cavernosal bodies to buttress the urethra.^7^ In 2002, Guralnick et al^8^ described the transcorporal technique. It consists of the placement of the AUS cuff through the corporal bodies in order to ensure enough tissue surrounding the urethra that could decrease the risk of erosion. Cavernosal tunica albuginea gives enough support to the dorsal aspect of the urethra, where the spongiosum is thinner. It also allows cuff placement without mobilizing the urethra, and therefore, it reduces the risk of further devascularization. Several series have described satisfactory results with this technique in the last 20 years.^8-10^ However, despite the reinforcement of the dorsal spongiosum, there is still certain risk of erosion ranging from 6.25% to 13%.^9,10^ Aaronson et al^9^ reported that the only erosion in the transcorporal technique group appeared at the ventral aspect of the urethra. Therefore, the implantation of a penile prosthesis in patients who had undergone this procedure may have a higher risk of complications, so patients willing to resume sexual intercourse in the future might not be the best candidates for this approach.

In view of these limitations, in 2019, a novel variant was described by Chouhan et al.^6^ consisting of the use of cavernosal tunica albuginea flaps from both corpora cavernosa in order to surround the atrophic spongiosum. After the flaps are obtained, a graft is used to cover the cavernosal defect, which theoretically would allow future penile prosthesis implantation.

This technical modification reinforces the ventral and lateral aspects of the urethra, while the dorsal part is covered by the intact part of the cavernosal tunica albuginea as in the standard transcorporal procedure. Although no case series with follow-up has yet been published, this is a promising technique.

Our case was a high-risk patient due to previous AUS, urethroplasty, and radiation therapy, so an additional technique was performed at the time of the second AUS implant in order to decrease the risk of complications. The election of the Gullwing modification technique was made in order to further reinforce the ventral and lateral aspects of the spongiosum. This complex technique must always be performed in centers with experience in reconstructive urethral surgery.

We are aware that this is only 1 case report and additional studies need to be done in order to prove the safety and efficacy of the technique. However, our follow-up of more than 24 months adds some evidence to the scarce literature currently available. 

## Conclusion

Transcorporal AUS placement is a suitable option in patients with high risk of urethral complications, but it still carries a certain risk of erosion, especially at the ventral aspect of the urethra, and may impact future erectile restoration surgery. Gullwing modification of the transcorporal technique is a novel adaptation that further reinforces the urethra at its ventral aspect and may decrease the risk of future penile prosthesis implantation procedures. Larger studies are needed in order to prove its utility in high-risk patients.
